# The Relationship between Social Support and Anxiety Symptoms in Informal Carers: A Systematic Review and Meta-Analysis

**DOI:** 10.3390/jcm12031244

**Published:** 2023-02-03

**Authors:** Emilia Priego-Cubero, Vasiliki Orgeta, Catalina López-Martínez, Rafael del-Pino-Casado

**Affiliations:** 1 Department of Nursing, Faculty of Health Sciences, University of Jaén, 23071 Jaén, Spain; 2 Division of Psychiatry, University College London, London W1T 7BN, UK

**Keywords:** anxiety symptoms, social support, perceived social support, caregiving, meta-analysis

## Abstract

Background: Providing care can be challenging for informal carers and increases the risk of mental health problems, such as experiencing clinical symptoms of anxiety. While strengthening social support for informal carers is a common recommendation to reduce this risk, no systematic review or meta-analysis to date has examined the relationship between social support and anxiety symptoms in informal carers. The aim of our study was to systematically review the current evidence on the association between perceived and received social support and anxiety symptoms in informal carers of dependent adults and older people, and to comment on the quality of the evidence. Methods: We searched PubMed, CINAHL (EBSCO), PsycINFO (ProQuest), Scopus, and LILACS up to 31 March 2021 for articles reporting on the association between caregiver anxiety symptoms and social support. A random-effects model was used to pool estimates, and each study was rated for quality using pre-specified criteria. Publication bias was assessed using a funnel plot and Egger’s regression test, which was adjusted using trim and fill analysis. Results: From the 2180 identified articles, 35 studies met our inclusion criteria, reporting on 5036 informal carers in total. We found a moderate negative association between perceived social support and caregiver anxiety symptoms (r = −0.31, 95% CI = −0.35, −0.27) and a small, negative association between received social support and caregiver anxiety (r = −0.15, 95% CI = −0.22, −0.08). Conclusion: The levels of perceived social support showed a significant negative association with caregiver anxiety symptoms. Policymakers and those working directly with informal carers should consider the development of targeted social support interventions that specifically enhance the levels of perceived social support to reduce symptoms of anxiety in informal carers.

## 1. Introduction

Increased life expectancy worldwide means that a large number of people currently live with chronic and disabling conditions that increase their dependency on others, such as family members and friends [1]. Informal carers often provide many hours of daily care, assisting with activities of daily living and medical tasks, which often involves providing high-intensity care. Caring for a dependent relative is stressful and has negative consequences for carers’ physical and psychological health [2,3]. Although caregiving may be positive and satisfactory for caregivers’ well-being [4], several decades of research have shown that informal carers experience high levels of subjective burden as a result of providing care [5,6,7] and are at increased risk of mental health problems, such as anxiety and depression [5,6,8]. 

Much of the caregiving literature over the last few decades has focused on caregiver burden and depression as primary outcomes, with fewer studies focusing on caregiver anxiety [9]. This is despite evidence indicating that most carers who experience depression also experience comorbid anxiety symptoms [10]. Although experiencing high levels of anxiety occasionally may not be harmful, severe and persistent anxiety represents a psychiatric condition, that can disrupt everyday functioning and decrease caregivers’ quality of life [11]. Several systematic reviews have reported that anxiety symptoms are highly prevalent across caregiving populations, such as carers of people surviving stroke [5], cancer [7], and carers of people living with Alzheimer’s disease [6]. Experiencing high levels of anxiety can lead to poor physical health outcomes for carers and directly impact care recipients; therefore, early identification and detection of factors that increase risk are key for future prevention and effective treatments [12]. 

### Social Support and Caregiving

Several theoretical models have shown that social support is a complex phenomenon that goes beyond the mere number of people in someone’s support network; social support is best described as a multidimensional construct that is dynamic, comprised several domains (structure of the support network, closeness of contacts, quality of relationships, and role of support), influenced by context, that may, in fact, change throughout our life course [13]. Both theory and empirical work have shown that social support can influence individuals’ psychological health, providing a useful framework for understanding how social networks may protect individuals under times of stress [13,14,15]. Social support has been defined as the actual or available social resources in times of need available for individuals that are perceived as positively supportive [16]. It can be further categorised as perceived (subjective or intangible) versus received social support (real or tangible) [17], and may comprise different dimensions, such as emotional (expression of thoughts, feelings, or needs), instrumental (physical support and tangible help), or informational support (direct informational advice or guidance) [18,19]. 

Recent theory and research in the area have highlighted that different types of social support may be differentially related to well-being, with perceived, rather than received, social support being a key contributor to psychological health [18,20]. In line with the transactional stress theory of Lazarus and Folkman [14], the negative consequences of caregiving may be mitigated by carers’ access to resources, such as social support. High levels of social support, for example, may improve carers’ positive interactions, reduce caregiver distress, and assist carers in coping with stressful events, which may directly decrease psychological distress [21,22,23]. 

Several studies have investigated whether social support has a direct effect on carers’ emotional health [18,24,25], with most studies to date [26] reporting on a significant association between levels of social support and anxiety symptoms in carers. However, no systematic review and meta-analysis to date have been conducted to collate individual studies and comment on the quality of evidence. As a result, the strength of the association between levels of social support and caregiver anxiety symptoms currently remains unknown, which makes the available evidence less accessible to decision-makers. 

The purpose of our study, therefore, was to systematically review current evidence on the association between perceived and received social support and anxiety symptoms in informal carers of dependent adults and older people, and comment on the quality of the evidence. Our secondary objective was to examine whether the magnitude of the association may differ between the two types of social support.

## 2. Materials and Methods

### 2.1. Design

For this systematic review and meta-analysis, we followed the recommendations of the Cochrane handbook [27], PRISMA [28], and MOOSE [29] guidelines, and registered our review with PROSPERO [30,31] (International Prospective Register of Ongoing Systematic Reviews) (Id.: CRD42021227287).

### 2.2. Search Strategy 

We searched PubMed, CINAHL (EBSCO), PsycINFO (ProQuest), Scopus, and LILACS using the following search terms: caregivers, social support, and anxiety, up to 31 March 2021, using an open search, without filters, to maximize sensitivity. We contacted, by mail, authors working in the subject area, which allowed us to locate grey literature and research that has not yet been published, thereby accessing data that did not appear in the published articles. We additionally searched the reference lists of relevant publications and reviews to ensure that no studies were missed. We applied no date or language limits to our search. 

### 2.3. Eligibility Criteria

The inclusion criteria for the selection of studies were: (a) original quantitative studies on informal carers of adults or older adults (≥18 years), (b) classifying social support as either perceived or received, (c) reporting on the association between anxiety symptoms and social support using a suitable statistic, such as a correlation coefficient (or another statistical parameter that could be transformed into a correlation coefficient), and € those that used a tool measuring all types of functional dimensions of social support (informational, instrumental, and emotional). Studies measuring only a specific functional dimension of social support were excluded to ensure that all studies used the same classification of social support and to limit heterogeneity.

Study selection was performed by two authors (RdPC and EPC). Each researcher independently applied the eligibility criteria to the title and abstract of each study, and if more information was required, the full-text article was located. Disagreements were resolved by discussion and reaching a consensus.

### 2.4. Data Extraction and Synthesis

Two authors independently extracted data (RdPC and EPC) using a standardized form, which was piloted before use and included information on the type of design, sample size, health/disease status of the care recipient, quality criteria, and effect sizes reported in each study. Disagreements were resolved through discussion until a consensus was reached.

### 2.5. Quality Assessment

The following criteria were used to assess the methodological quality of the individual studies [32,33]: (1) type of sampling (use of probability sampling or not); (2) validity and reliability of measurements used (content validity and internal consistency of questionnaires in the target population or similar), with this criterion being mandatory for a study to be included in the meta-analysis; (3) control for confounding factors (controlling for at least one measure of objective caregiver burden); and (4) for longitudinal studies, attrition (follow-up rate ≥ 80% of the original population participating in the study).

We chose objective caregiver burden as a key confounding factor to control for, due to its association with symptoms of anxiety [34]. As measures of objective caregiver burden are strongly intercorrelated [35], we rated as adequate all studies controlling for at least one measure of objective caregiver burden (activities of daily living of the care recipient, presence of cognitive impairment, neuropsychiatric symptoms experienced by the care recipient, or intensity of care provided by informal carers) in the design and/or analysis (e.g., through multivariate analysis) [32,36]. When statistical adjustment was performed, we considered confounding bias to be absent if the variation in the point estimate was less than 10% [37].

In line with the Grading of Recommendations Assessment, Development and Evaluation (GRADE) system [38], we additionally examined the risk of publication bias, inconsistency, and imprecision in each of the studies. Publication bias was evaluated by analysing the funnel plot and statistical tests. We assessed inconsistency by the presence of heterogeneity in the findings of individual studies and the imprecision of results by considering the number of included studies contributing to each meta-analysis (large: >10 studies, moderate: 5–10 studies and small: <5 studies), and the median sample size (high: >300 participants, intermediate: 100–300 participants, and low: <100 participants). Two authors (RdPC and CLM) conducted the quality assessment independently, and any discrepancy was resolved by discussion and reaching a consensus.

### 2.6. Analyses

We used the correlation coefficient as a measure of the estimate of the effect. In line with the recommendations of Cooper et al. [39], we used a random-effects model to combine results in our meta-analysis that would allow us to generalize findings across all caregiving groups. For longitudinal studies reporting repeated measures and cross-sectional correlations at each time point, the first correlation was taken into account. Heterogeneity was measured with Cochran’s Q (with *p* values > 0.10 indicating no evidence of heterogeneity) and the I² index, which measures heterogeneity due to variability in the effect estimate between individual studies in a meta-analysis (we considered the values of 25, 50, and 75% as indicative of mild, moderate, and high heterogeneity respectively). 

We used the Egger test [40] to assess publication bias, which statistically evaluates asymmetry evident in a funnel plot (a *p* value below 0.1 suggests asymmetry), and also used the Trim and Fill method [41] to calculate the combined effect adjusted for publication bias. We investigated the robustness of our results by conducting several sensitivity analyses; these included the leave-one-out method (eliminating one study at a time) and several subgroup analyses to examine whether the study design and study quality influenced the results. We used Comprehensive Meta-Analysis 3.1 for all analyses.

## 3. Results

### 3.1. Search Results

**Figure 1:**
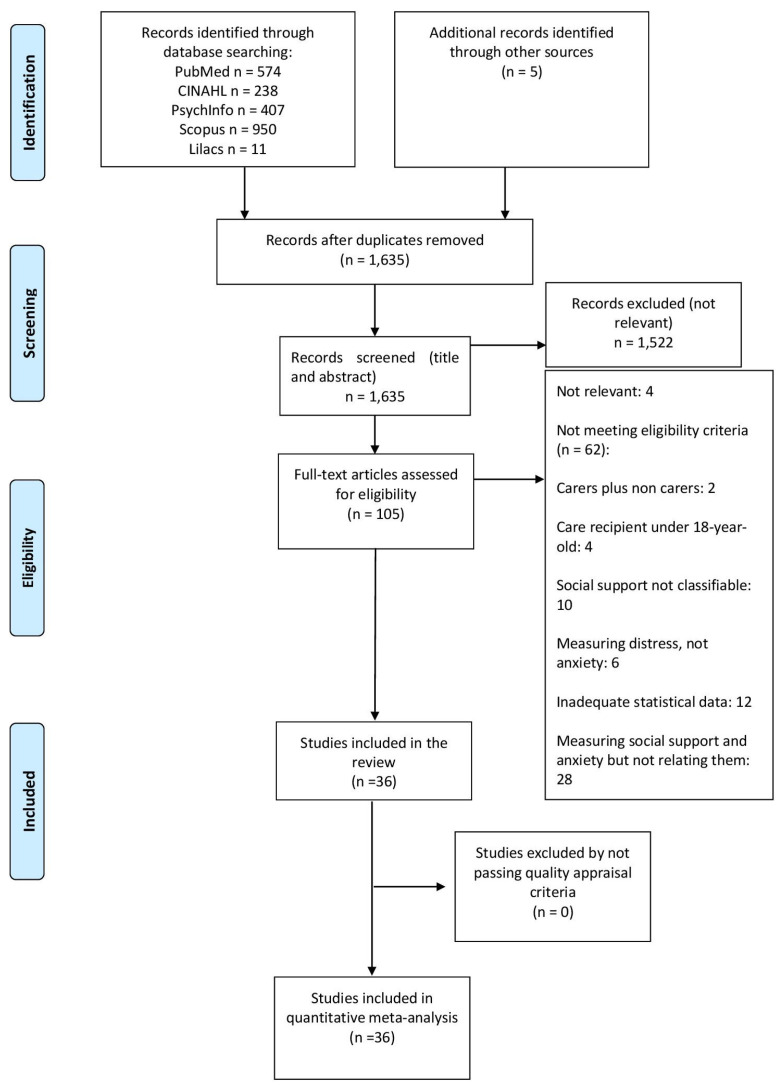
PRISMA flow diagram of the review process.

A total of 2180 records were retrieved from the databases, with 5 additional references identified via searching the references of included studies (Figure 1). After removing duplicates, 1635 records were screened, of which 1531 were discarded as not being relevant (not related to the subject of the study), leaving 104 records to be screened via full-text evaluation. Of these, 8 studies were excluded as not relevant and 62 studies as not meeting the inclusion criteria, leaving a total of 34 studies [26,42,43,44,45,46,47,48,49,50,51,52,53,54,55,56,57,58,59,60,61,62,63,64,65,66,67,68,69,70,71,72,73,74] meeting the inclusion criteria of the review. Studies were considered as not relevant and were excluded when their topic was not directly relevant to that of the present review (i.e., measured coping and anxiety symptoms in carers, but did not report data on their association).

### 3.2. Description of Included Studies

The characteristics of the included studies are presented in Table 1. Most studies were on informal carers of people living with cancer (eight studies), frail older people (seven studies), and people living with dementia (four studies). In the remaining studies, the cause of care dependency was stroke (three studies), traumatic brain injury (two studies), or other physical illness, with one study on informal carers of people living with mental illness. Twenty-seven studies used a cross-sectional design and seven used a longitudinal design with repeated measures. Among the seven longitudinal studies, five reported longitudinal correlations and two reported cross-sectional correlations (referring to the same time point).

### 3.3. Quality Assessment

Table 2 presents the quality ratings of each of the individual studies. All studies except for two used non-probability samples, and only five studies controlled for confounders. All longitudinal studies reported an absence of attrition. 

### 3.4. Results of the Meta-Analysis

The 34 included studies provided 34 samples with 35 independent comparisons (32 reporting data on perceived social support and 3 on received social support). 

#### 3.4.1. Perceived Social Support

Thirty-two studies (Table 3) reported on the association between perceived social support and anxiety symptoms, reporting on thirty-two independent samples with thirty-two independent comparisons. Most of these studies used non-probability samples (*n* = 30), reported on cross-sectional correlations (*n* = 28), and did not control for confounders (*n* = 27). The main care recipients were frail older adults (*n* = 7), people living with cancer (*n* = 7), and people living with dementia (*n* = 4).

**Figure 2:**
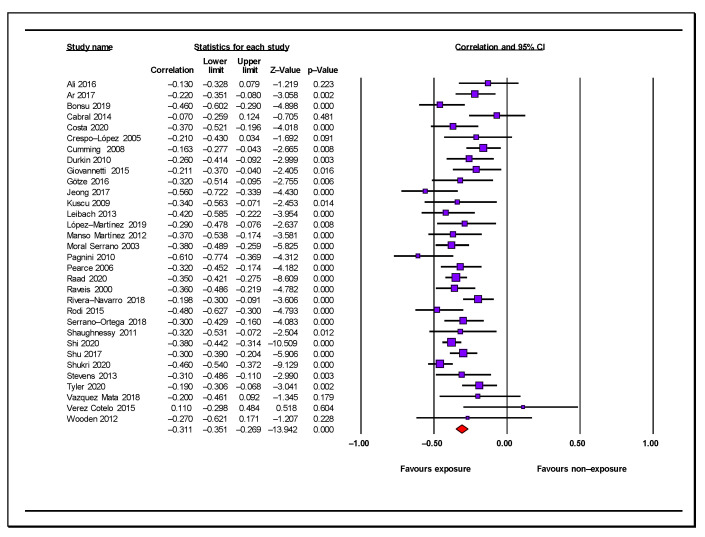
Forest plot for perceived social support and anxiety symptoms in informal carers [25,42,43,44,45,46,47,48,49,50,52,54,55,56,57,58,59,60,61,62,63,64,65,66,67,68,69,70,71,72,73,74].

**Figure 3:**
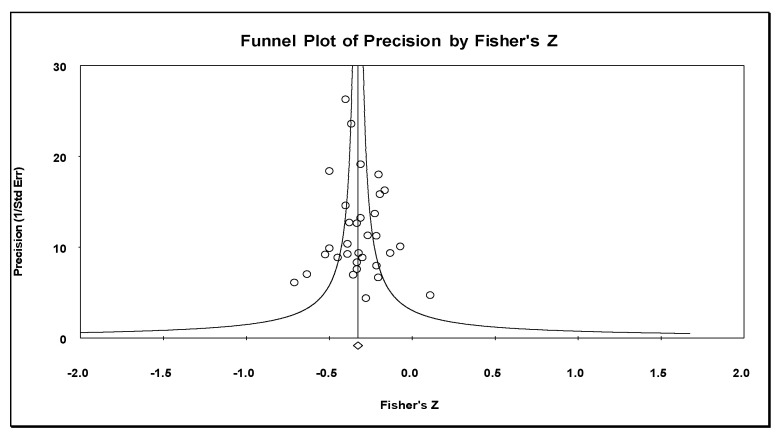
Funnel plot for perceived social support and anxiety symptoms in informal carers.

The combined effect (r = −0.31, 95% CI = −0.35, −0.27, *n* = 4970, mean sample size: 155.3) showed a moderate negative association between perceived social support and caregiver anxiety symptoms (Table 3; Figure 2). The effect was statistically significant in the individual samples, except for those in six studies, and all reported associations had a negative direction (Figure 2). We considered the results of our meta-analysis as precise due to the number of included studies and the mean sample size. There was evidence of low heterogeneity among individual studies (Q = 33.16 degree of freedom [gl] = 31, *p* = 0.36, I^2^ = 6.5%). Inspections of the funnel plot (Figure 3) showed that this was somewhat symmetrical, with the results of the Egger’s test (*p* = 0.56) being consistent, with no evidence of publication bias. The combined effect calculated by the Trim and Fill method (r = −0.31) did not vary from the original combined effect; therefore, the risk of publication bias was low.

Sensitivity analysis showed that removing one study at a time resulted in variations in the pooled estimate under 2.6%. Subgroup analyses showed no differences in the pooled estimate by the type of study design or quality criteria of the individual studies. Studies employing a longitudinal design (repeated measures studies with correlations referred to different time points) showed a similar pooled effect (r = −0.29; 95% CI = −0.37, −0.20; four samples) to the cross-sectional and repeated-measures studies reporting on cross-sectional correlations (r = −0.32; 95% CI = −0.34, −0.29; 28 samples). The pooled estimate from studies controlling for confounders (r = −0.35; 95% CI = −0.44, −0.25; five samples) was similar to that reported by studies not controlling for confounders (r = −0.30; 95% CI = −0.35, −0.26; 27 samples). There were no differences in the results when comparing studies that used a probability sample versus those that did not (studies with non-probability sampling: r = −0.31; 95% CI = −0.36, −0.27; 30 samples; studies with probability sampling: r = −0.30; 95% CI = −0.41, −0.18; two samples). 

#### 3.4.2. Received Social Support

**Figure 4:**
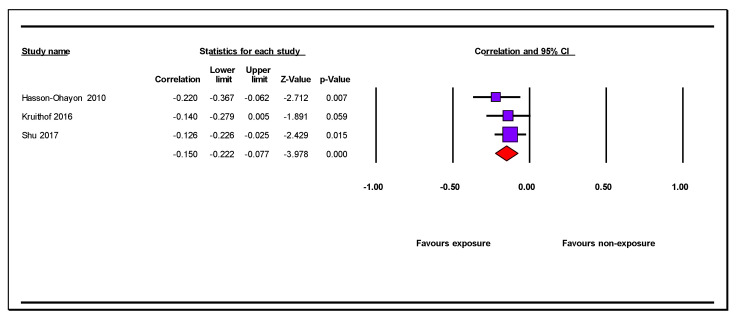
Forest plot of the association of received social support and caregiver anxiety symptoms [51,53,68].

Three studies (Table 1) reported on the association between received social support and caregiver anxiety symptoms. These studies included three independent samples with three independent comparisons, of which two were cross-sectional and one was longitudinal. All studies used a non-probability sample and did not control for potential confounders. Care recipients were frail older adults, people surviving stroke, and people living with cancer. The combined effect (r = −0.15, 95% CI = −0.22, −0.08, *n* = 526, mean sample size: 397.4) showed a small negative association between caregiver anxiety symptoms and received social support (Table 3; Figure 4). The associations among the individual samples were all negative (Figure 4). We considered the results of this meta-analysis as moderate to low in terms of precision due to the small number of studies and median sample size. There was no heterogeneity in the results (Q = 1.004 degrees of freedom [gL] = 2 *p* = 0.61, I^2^ = 0%). We were unable to perform an assessment of publication bias, nor subgroup analyses due to the low number of studies.

## 4. Discussion

This study provides the first systematic review and meta-analysis of the association between perceived and received social support and anxiety symptoms in informal carers. Our review finds that informal carers who report low levels of perceived social support are more likely to report experiencing high levels of anxiety symptoms. The results of our meta-analyses showed that this association represents a moderate effect overall, which remained robust after controlling for several potential biases. Our findings indicate that informal carers who report low levels of perceived social support may be more vulnerable to experiencing high levels of anxiety [22]. Although fewer studies examined the association between received social support and anxiety symptoms, our meta-analyses showed that low levels of received social support may also constitute an important marker of caregiver anxiety symptoms. Collectively, our results support previous findings of the buffer effect of social support, known as the stress-buffering hypothesis, whereby social support weakens the effect of stress on mental health outcomes [75].

We can be relatively confident about the robustness of our results quantifying the association between perceived support and caregiver anxiety symptoms due to the large number of studies included in our meta-analysis, the low heterogeneity observed, and the absence of publication bias. Our quantitative synthesis was based on studies reporting on large sample sizes that were conducted in different caregiving groups, which increased the generalizability of our results. An important strength of our findings is that most of the studies included used measures with high reliability and validity, increasing our confidence in the results. We found no differences in our subgroup analyses between studies that employed non-probability sampling versus those that did not, and the size of the association remained the same between studies controlling for confounders versus those that did not, indicating that these factors were less likely to have influenced the results.

Our findings are, overall, consistent with both theory and empirical work in the area [18,24,25], showing that perceived social support is an important resource for informal carers, promoting adjustment to the caregiving role [24], and being protective of psychiatric distress [18,25]. Perception of social support as adequate by informal carers is, therefore, an important determinant of their psychological health and may support carers in perceiving the caregiving situation as less stressful, thereby decreasing their risk of experiencing high levels of anxiety [15,21]. These findings point towards the importance of healthcare professionals discussing and monitoring the levels of perceived social support in carers in clinical settings and signposting family members to relevant available resources. Future studies, however, are required to understand how levels of perceived social support can be enhanced in the context of support interventions for informal carers and the factors that influence these perceptions.

Despite our analyses showing that caregiver anxiety symptoms were also negatively associated with their received levels of social support, our confidence in this finding remains low, as the number of studies contributing to our meta-analysis was very small. It will be important for future work in the area to conduct large-scale studies examining the relationship between received social support and anxiety symptoms in informal carers and understanding which factors may influence this association. Although preliminary, our findings do suggest that perceived, rather than received, social support may be central to anxiety symptoms in carers and may reduce anxiety over time [16,20].

An important contribution of our review is that it improves our understanding of the differential contributions of perceived versus received social support on mental health outcomes for carers [18,76] and how this knowledge may be used in preventing and treating these distressing symptoms. Given the important distinction between the perceived and received levels of social support, our results indicate that interventions aimed at improving perceived social support could be more effective in improving the psychological well-being of informal carers than those targeting received support. Policymakers and relevant key stakeholders should, therefore, consider commissioning the development of interventions that aim to increase perceptions of social support, rather than the quantity of support received.

### Limitations

Despite several strengths, our review has several limitations. First, the majority of the studies included in our review were cross-sectional, with very few longitudinal studies contributing to our meta-analysis. Nevertheless, our subgroup analyses indicated that the type of study design had little effect on the association between perceived social support and carer anxiety symptoms. Second, most of the studies used convenience samples and only a very small number of studies controlled for potential confounders. However, we found no differences in our subgroup analyses examining the influence of the type of sampling method and whether the studies controlled for confounders.

Reverse causation is possible between low levels of social support and higher anxiety symptoms in carers; therefore, to address this issue, future longitudinal studies are needed. An important limitation is that we were also not able to control for important confounders affecting the relationship between social support and anxiety symptoms, such as stressful life events. We were also not able to assess the influence of several socio-economic factors and their effects on caregiver anxiety symptoms. The assessment measures used for social support and anxiety could be susceptible to measurement bias, and they were primarily based on self-report instruments, which limits the results of our analyses. Our second meta-analysis quantifying the association between received social support and anxiety symptoms was limited by the very small number of studies and should, therefore, be interpreted with caution.

## 5. Conclusions

Despite these limitations, our study contributes important new evidence to understanding the association between social support and caregiver anxiety symptoms. Perceived social support is an important factor affecting anxiety symptoms in carers, which may be protective in the long term. Future longitudinal studies are needed to improve our understanding of the causal relationship between social support and anxiety symptoms in caregiving populations and to investigate the most important factors contributing to this association.

## Figures and Tables

**Table 1:** Description of studies included in the review.

Studies (Author, Year) Country	*n*	Measure of Social Support	Measure of Anxiety	Design	Care Recipients
Ali, 2016 [42] Pakistan	90	MSPSS	DASS	Cross-sectional	Stroke
Ar, 2017 [43] Turkey	190	MSPSS	STAI	Cross-sectional	Dementia
Bonsu, 2019 [44] Ghana	100	MSPSS	BAI	Cross-sectional	Severe burns injury
Cabral, 2014 [45] Portugal	104	ESSS	EADS	Cross-sectional	Mental illness
Costa, 2020 [26] Portugal	110	SSSS	DASS	Cross-sectional	Diabetes
Crespo López, 2005 [46] Spain	66	SSSQ	HADS	Cross-sectional	Dementia and older people without dementia
Cumming, 2008 [47] Australia	174	MOS-SS	IDA	Cross-sectional (^1^)	Stroke
Durkin, 2010 [48] U.S.A.	130	ISEL-6	STAI	Repeated measures	Frail older adults
Giovannetti, 2015 [49] Italy	129	MOS-SS	STAI	Cross-sectional	Disorders of consciousness
Götze, 2016 [50] Germany	72	OSS-3	HADS	Repeated measures	Cancer
Hasson-Ohayon, 2010 [51] Israel	150	CPASS	BSI	Cross-sectional	Cancer
Jeong, 2017 [52] South Korea	52	DUKE-UNC	HADS	Cross-sectional	Cancer
Kruithof, 2016 [53] Netherlands	183	SSL-12-I	HADS	Repeated measures	Stroke
Kuscu, 2009 [54] Turkey	51	MSPSS	STAI	Cross-sectional	Cancer
Leibach, 2013 [55] Mexico	81	ISEL-12	STAI	Cross-sectional	Multiple sclerosis
López-Martínez, 2019 [56] Spain	81	DUKE-UNC	GAS	Repeated measures	Frail older adults
Manso Martínez, 2012 [57] Spain	88	DUKE-UNC	HADS	Cross-sectional	Frail older adults
Moral Serrano, 2003 [58] Spain	215	DUKE-UNC	GAS	Cross-sectional	Frail older adults
Pagnini, 2010 [59] Italy	40	MG-SS	STAI	Cross-sectional	Amyotrophic lateral sclerosis
Pearce, 2006 [60] U.S.A.	162	ISEL	SCID	Cross-sectional	Cancer
Raad, 2020 [61] U.S.A.	558	TBI-CareQOL	TBI- CareQOL	Cross-sectional	Traumatic brain injury
Raveis, 2000 [62] U.S.A.	164	ISEL	STAI	Cross-sectional	Cancer
Rivera-Navarro, 2018 [63] Spain	326	DUKE-UNC	HADS	Cross-sectional	Dementia
Rodi, 2015 [64] U.S.A.	87	MOS-SS	BAI	Cross-sectional	Cancer
Serrano-Ortega, 2018 [65] Spain	177	DUKE-UNC	GAS	Repeated measures	Frail older adults
Shaughnessy, 2011 [66] Canada	60	MOS-SS	STAI	Cross-sectional	Mild cognitive impairment
Shi, 2020 [67] China	693	MSPSS	SAS	Cross-sectional	Frail older adults
Shu, 2017 [68] Australia	193	DSSI-Short	GAS	Cross-sectional	Frail older adults
Shukri,2020 [69] Malaysia	340	MSPSS	HADS	Cross-sectional	Haemodialysis patients
Stevens, 2013 [70] Mexico	90	ISEL	STAI	Cross-sectional	Traumatic brain injury
Tyler, 2020 [71] Mexico	253	ISEL	GAD	Cross-sectional	Parkinson’s disease
Vazquez Mata, 2018 [72] Mexico	47	DUKE-UNC	HADS	Cross-sectional (^1^)	Cancer
Verez Cotelo, 2015 [73] Spain	25	DUKE-UNC	STAI	Cross-sectional	Dementia
Wooden, 2012 [74] U.S.A.	22	MSPSS	POMS	Cross-sectional	Dementia

Notes: (^1^): the study is longitudinal with repeated measures, but the correlations are based on the same time point. Note: Abbreviations of the measures are presented in Appendix A.

**Table 2:** Quality assessment of the included studies.

Studies	Probabilistic Sampling	Reliability and Validity of Measures (Mandatory)	Control of Confounders	Absence of Attrition
Ali, 2016 [42]	-	+	?	N/A
Ar, 2017 [43]	-	+	?	N/A
Bonsu, 2019 [44]	-	+	?	N/A
Cabral, 2014 [45]	-	+	?	N/A
Costa, 2020 [26]	-	+	?	N/A
Crespo López, 2005 [46]	-	+	?	N/A
Cumming, 2008 [47]	-	+	?	N/A
Durkin, 2010 [48]	-	+	?	+
Giovannetti, 2015 [49]	-	+	?	N/A
Götze, 2016 [50]	-	+	-	N/A
Hasson-Ohayon, 2010 [51]	-	+	?	N/A
Jeong, 2017 [52]	-	+	?	N/A
Kruithof, 2016 [53]	-	+	?	+
Kuscu, 2009 [54]	-	+	?	N/A
Leibach, 2013 [55]	-	+	-	N/A
López-Martínez, 2019 [56]	+	+	+	+
Manso Martínez, 2012 [57]	-	+	+	N/A
Moral Serrano, 2003 [58]	-	+	-	N/A
Pagnini, 2010 [59]	-	+	+	N/A
Pearce, 2006 [60]	-	+	?	N/A
Raad, 2020 [61]	-	+	?	N/A
Raveis, 2000 [62]	-	+	-	N/A
Rivera-Navarro, 2018 [63]	-	+	?	N/A
Rodi, 2015 [64]	-	+	?	N/A
Serrano-Ortega, 2018 [65]	+	+	+	+
Shaughnessy, 2011 [66]	-	+	?	N/A
Shi, 2020 [67]	-	+	?	N/A
Shu, 2017 [68]	-	+	?	N/A
Shukri,2020 [69]	-	+	?	N/A
Stevens, 2013 [70]	-	+	+	N/A
Tyler, 2020 [71]	-	+	?	N/A
Vazquez Mata, 2018 [72]	-	+	?	N/A
Verez Cotelo, 2015 [73]	-	+	?	N/A
Wooden, 2012 [74]	-	+	?	N/A

Abbreviations: N/A: not applicable.

**Table 3:** Summary of the meta-analysis results.

	Studies	Samples	*n*	Mean per Sample	*r*	95% CI		I^2^	Publication Bias			
						Lower	Upper		Funnel Plot	Egger’s Test *p*-Value	Trim and Fill	
											Estimate	Variation
Perceived social support	32	32	4970	155.3	−0.31	−0.35	−0.27	6.5%	Asymmetric	0.65	−0.31	0.0
Received social support	3	3	526	397.4	−0.15	−0.22	−0.08	0%	Asymmetric	0.4	−0.15	0.0

Abbreviations: *r*: combined correlation coefficient, CI: confidence interval, I^2^: degree of inconsistency.

## Data Availability

The data presented in this study are available on request from the corresponding author.

## Appendix A

**Table A1:** Abbreviations of Measures.

**Social Support**	
CPASS	Cancer Perceived Agents of Social Support
DSSI-Short	Duke Social Support Index (short version)
DUKE-UNC	Duke-UNK functional social support questionnaire
ESSS	Satisfaction with Social Support Scale
ISEL	Interpersonal Support Evaluation List
MG-SS	Social support subscale of the McGill Quality of Life Questionnaire
MSPSS	Multidimensional Scale of Perceived Social Support
MOS-SS	Medical Outcomes Study—Social Support Scale
OSS-3	Oslo Social Support Scale
SSL-12-I	Social Support List—Interaction
SSSQ	Sarason Social Support Questionnaire
SSSS	Satisfaction with Social Support Scale
TBI-CareQOL	Social support factor of the Traumatic Brain Injury Caregiver Quality of Life
**Anxiety**	
BAI	Beck Anxiety Inventory
BSI	Brief Symptom Inventory
DASS	Depression Anxiety Stress Scale
EADS	Anxiety, Depression and Stress Scale
GAD	Generalized Anxiety Disorder Assessment
GAS	Goldberg Anxiety Scale
HADS	Hospital Anxiety Depression Scale
HARS	Hamilton Anxiety Rating Scale
IDA	Irritability, Depression, and Anxiety Scale
SCID	Structured Clinical Interview for the DSM-IV
POMS	Profile of Mood States
SAS	Self-Rating Anxiety Scale
STAI	State Trait Anxiety Inventory
TBI-CareQOL	Anxiety factor of the Traumatic Brain Injury Caregiver Quality of Life
